# Insights on microstructural evolution and capacity fade on diatom $\hbox {SiO}_2$ anodes for lithium-ion batteries

**DOI:** 10.1038/s41598-023-47355-7

**Published:** 2023-11-22

**Authors:** Weicheng Hua, Inger-Emma Nylund, Federico Cova, Ann Mari Svensson, Maria Valeria Blanco

**Affiliations:** 1 Department of Materials Science and Engineering, Norwegian University of Science and Technology, NO-7491 Trondheim, Norway; 2 BL31 FaXToR Beamline, CELLS- ALBA Synchrotron Light Source, 08290 Cerdanyola del Vallès, Barcelona Spain

**Keywords:** Batteries, Batteries

## Abstract

$\hbox {SiO}_2$ is a promising material for developing high-capacity anodes for lithium-ion batteries (LIBs). However, microstructural changes of $\hbox {SiO}_2$ anodes at the particle and electrode level upon prolonged cycling remains unclear. In this work, the causes leading to capacity fade on $\hbox {SiO}_2$ anodes were investigated and simple strategies to attenuate anode degradation were explored. Nanostructured $\hbox {SiO}_2$ from diatomaceous earth was integrated into anodes containing different quantities of conductive carbon in the form of either a conductive additive or a nanometric coating layer. Galvanostatic cycling was conducted for 200 cycles and distinctive trends on capacity fade were identified. A thorough analysis of the anodes at selected cycle numbers was performed using a toolset of characterization techniques, including electrochemical impedance spectroscopy, FIB-SEM cross-sectional analysis and TEM inspections. Significant fragmentation of $\hbox {SiO}_2$ particles surface and formation of filigree structures upon cycling are reported for the first time. Morphological changes are accompanied by an increase in impedance and a loss of electroactive surface area. Carbon-coating is found to restrict particle fracture and to increase capacity retention to 66%, compared to 47% for uncoated samples after 200 cycles. Results provide valuable insights to improve cycling stability of $\hbox {SiO}_2$ anodes for next-generation LIBs.

## Introduction

Since their commercialization 30 years ago, LIBs have become the dominant technology in portable electronics^1^. Moreover, Li-ion technology dominance will be further emphasized by the electrification of the vehicle fleet, as the number of electric vehicles is expected to increase by 2–3 orders of magnitude by 2040^2^. The expected increase in battery demand will require the development of more energy-dense materials with high rate-capabilities. The primary anode material in current state-of-the-art Li-ion technologies is graphite, as it displays excellent cycling stability, low working potential and relatively low-cost and abundance^3–5^. However, graphite’s maximum theoretical capacity of ${372}\,\hbox {mAhg}^-1$ is a limiting aspect in designing high energy density batteries for future applications.

Silicon (Si) has emerged as one of the most promising graphite alternatives due to its high theoretical capacity and widespread availability^6^. During electrochemical cycling, Si reversibly reacts with lithium to form a $\hbox {Li}_\text{x}\hbox {Si}$ alloy and is capable of reaching specific capacities of ${3590}\,\hbox {mAhg}^-1$ for the $\hbox {Li}_{15}\hbox {Si}_4$ phase. However, practical application of Si anodes is still held back by several major challenges. These include large volume variations (up to 400%) between lithiation and delithiation, poor electrical conductivity, and an inability to form a stable solid electrolyte interphase (SEI). The volume variations induce significant mechanical stresses within the anode, leading to potential particle pulverization and subsequent loss of electrical contact with conductive additive or the current collector. The SEI is also constantly fractured as the underlying particle expands and contracts, and the SEI is continuously reformed. This results in the continuous consumption of liquid electrolyte, increased impedance and capacity fade^7^. These challenges have to do with severe irreversible capacity fade commonly found in silicon anodes. Several methods have therefore been developed to improve the cycling stability of Si anodes, and among them intricate nano-structuring and extensive engineering of coatings, binders, and conductive additives have shown beneficial effects^8^. In particular, coating Si particles with a thin nanometric layer of $\hbox {SiO}_2$ has been hypothesized to limit volume expansion by inducing hydrostatic compressive stresses that will shift the equilibrium concentration of Li in the formed $\text{Li}_\text{x}\text{Si}$ phase towards lower values^9–11^.

Interestingly, $\hbox {SiO}_2$ was originally thought to be electrochemically inert against $\hbox {Li}^+$, due to its low electrical conductivity and Li-ion diffusivity^12,13^. However, later studies found that $\hbox {SiO}_2$ nanoparticles can react with Li via reduction reactions^14,15^. The electrochemical reactions between $\hbox {SiO}_2$ and Li-ions form both reversible and irreversible products and are listed below alongside the potential they occur at vs. $\hbox {Li}^+$/Li^16^:

1. $$4\hbox {Li}^{+} + 4\hbox {e}^{-} + 5\hbox {SiO}_{2} <-> 2\hbox {Li}_{2}\hbox {Si}_2\hbox {O}_{5}$$ + Si   1.3V
2. $$4\hbox {Li}^{+} + 4\hbox {e}^- + 3\hbox {SiO}_2 -> 2 \hbox {Li}_2\hbox {SiO}_3$$ + 2Si    $$\approx$$ 1.15-1.25V
3. $$4\hbox {Li}^{+} + 4\hbox {e}^- + 2\hbox {SiO}_2 -> \hbox {Li}_4\hbox {SiO}_4$$ + Si    $$\approx$$ 1.15-1.25V
4. $$4\hbox {Li}^{+} + 4\hbox {e}^- + \hbox {SiO}_2 -> 2\hbox {Li}_2\hbox {O}$$ + Si    0.7V

R1 is the most thermodynamically favourable reaction and forms the only reversible lithium silicate product ($\hbox {Li}_2\hbox {Si}_2\hbox {O}_5$)^16^. Formation of $\hbox {Li}_2\hbox {O}$ occurs at the lowest potential below $\approx$ 0.7V vs. $\hbox {Li}^+$/Li. Large overpotentials due to kinetic limitations are commonly observed experimentally, and it is generally accepted that a combination of $\hbox {Li}_2\hbox {Si}_2\hbox {O}_5$, $\hbox {Li}_4\hbox {SiO}_4$, $\hbox {Li}_2\hbox {O}$ and $\hbox {Li}_\text{X}\hbox {Si}$ are formed during lithiation. The accurate reaction routes are not clearly identified due to huge differences in electrochemical behaviour depending on the particle and electrode properties of the studied systems^17–19^. The Si nano-domains formed from $\hbox {SiO}_2$ lithiation will proceed to alloy reversibly with Li-ions and is the main contributor towards the reversible capacity^20,21^. During electrochemical cycling, $\hbox {SiO}_2$ undergoes in situ formation of complex nanocomposites consisting of both electrochemically active silicon and electrochemically inactive lithium silicates. The inactive domains have been speculated to act as a mechanical scaffold which limits volume variations to $\approx$ 100%^12^. $\hbox {SiO}_2$ therefore allows for the design of electrodes with good cycling stability while also having a relatively large lithium ion storage capacity (${1950}\,\hbox {mAhg}^-1$), low cost and high global abundance. Improvements to $\hbox {SiO}_2$ performance can be achieved by employing conductive carbon as a coating matrix that not only improves electronic conductivity, but also helps alleviate stresses associated with Li-ion insertion and extraction^22–26^.

The performance of silica anodes is highly dependent on their design and structure down to the particle level. Nanostructuring has been found to improve anode performance by reducing diffusion paths of Li-ions and by accommodating volume variations^27^. $\hbox {SiO}_2$ and $\hbox {SiO}_2$/C in the form of nanoparticles, nanotubes and nanofiber have all been previously studied^28,29^. A natural source for nanostructured $\hbox {SiO}_2$ can be found in the shells of microscopic single-celled algae called diatoms. Diatoms grow rigid cell walls (frustules), composed of amorphous silica, with highly ordered pore structures which are species-specific, and display unique mechanical and molecular transport properties^30–34^. Though diatoms can be easily cultivated in large quantities, an even cheaper source of diatom silica is diatomite or diatomaceous earth (DE) formed by fossilisation of diatoms that lived millions of years ago. DE mineral is available as a cheap material in millions of tons from the mining industry^30^.

As reported by Lisowska et al.^35^, $\hbox {SiO}_2$/C composite anodes obtained through pyrolysis of red algae covered by diatoms achieved capacities of ${500}\,\hbox {mAhg}^-1$ over 80 cycles and a capacity retention of 94%. Nowak et al.^36^ achieved capacities of ${521}\,\hbox {mAhg}^-1$ with a capacity retention of 97% through coating of the diatoms using the native organic matter to obtain a crystalline $\hbox {SiO}_2$/C composite. Nowak et al.^37^ later showed that anodes containing a mixture of diatom biosilica and carbon black in a 1:1 ratio display a specific capacity of ${400}\,\hbox {mAhg}^-1$ over 90 cycles. The capacity fade that was observed for anodes made with biosilica to carbon black ratios of 1:2, was associated with crack formation on the surface of the cycled electrodes. Previous work has also involved carbon-coating of *Coscinodiscus* diatoms via pyrolysis of sucrose at 650$^\circ$C before fabricating an electrode containing 35 wt% carbon black^38^. In this work, a novel potentiostatic lithiation step at low voltages was introduced to force the highest capacity generating reaction, (R4) in the reaction table above, to occur and ensure high degrees of $\hbox {SiO}_2$ conversion to Si. This resulted in a high reversible capacity of ${723}\,\hbox {mAhg}^-1$ for 50 cycles^38^. In a posterior work, Blanco et al.^39^ optimized carbon-coating parameters on $\hbox {SiO}_2$ electrodes by looking at different precursor solutions of glucose, sucrose and cornstartch at different concentrations and found that pyrolysis of 40 wt% sucrose at 1200$^\circ$C significantly improves capacity retention in non-porous $\hbox {SiO}_2$ . When using DE nanoporous $\hbox {SiO}_2$, pyrolysis treatment at 850$^\circ$C of the sucrose precursor has shown to successfully increase cycling stability^40^. In a more recent work, Wang et al.^41^ investigated three different species of cultured diatoms as $\hbox {SiO}_2$ sources as well as the effect of calcining the surrounding biomass as a source of conductive carbon. This resulted in half-cells achieving capacities of $\approx$ ${1000}\,\hbox {mAhg}^-1$ for 200 cycles at a current density of 400$\hbox {mAg}^-1$. Campbell et al.^42^ used DE $\hbox {SiO}_2$ as the source for fabricating nanosilicon electrode through magnesiothermic reduction. The DE-based nano-Si anode displayed good cyclability with a specific capacity of $\approx$ ${1100}\,\hbox {mAhg}^-1$ after 50 cycles.

Nanostructured $\hbox {SiO}_2$ is gaining increasing attention as an anode material for next-generation Li-ion batteries, and diatom $\hbox {SiO}_2$ is a possible sustainable source for obtaining naturally nanostructured material. However, there is a lack of insight on such electrode material after the cycling capacity starts to fade and on the underlying mechanism behind it, in particular on their microstructural changes. Indeed, to the author’s knowledge, there is only one research work in the existing literature on diatom $\hbox {SiO}_2$ anodes that has reported electrochemical cycling data above 100 cycles. In this work, a comprehensive investigation of capacity fade mechanisms in diatom-based $\hbox {SiO}_2$ anodes as well as the effect of conductive additives and carbon coating on capacity retention were investigated. Electrodes containing both $\hbox {SiO}_2$ and $\hbox {SiO}_2$/C composites with different amounts of conductive carbon black were fabricated. Electrochemical performance is evaluated through galvanostatic cycling as well as Electrochemical Impedance Spectroscopy (EIS). Morphological evolution of the system at the electrode and particle level is then evaluated through FIB-SEM cross section analysis and TEM inspections. This study provides a valuable understanding on the causes leading to capacity fade of $\hbox {SiO}_2$ anodes and will be beneficial for the design of improved $\hbox {SiO}_2$-based materials for anodes of next-generation LIBs.

## Results

### Characterization of diatomaceous earth $\hbox {SiO}_2$ frustules

**Figure 1:**
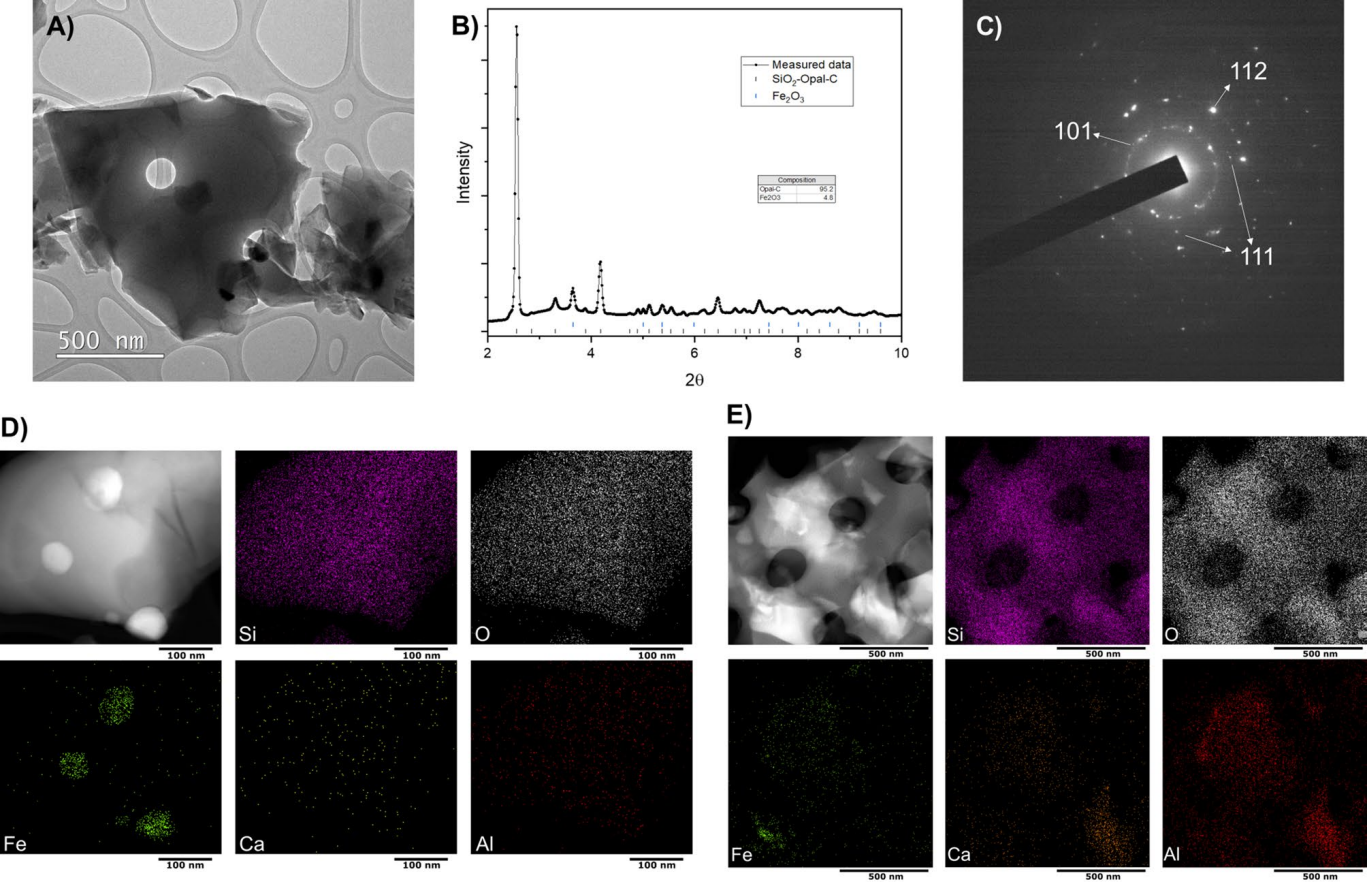
(**A**) Bright-field TEM images of diatomaceous earth frustules after ball-milling, (**B**) Synchrotron powder X-ray diffractogram of milled diatom frustules, (**C**) SAD of diatom frustules, (**D**) and (**E**) STEM-EDX image of two different fragments of a milled diatom frustule showing elemental chemical mappings of Si, O, Fe, Ca and Al.

Figure 1A depicts a bright-field TEM image of milled $\hbox {SiO}_2$ frustules. Characteristic features of frustule nanostructure are clearly visible as shown by the presence of circular cavities on the frustule. Interestingly, there are regions with high contrast, which can be attributed to the presence of denser materials other than $\hbox {SiO}_2$. These contrasts can be seen in the HAADF-STEM images in Fig. 1D and e as well. To determine all possible crystallographic phases present in the frustule, synchrotron X-ray powder diffraction data was collected. The obtained diffractogram is shown in Fig. 1B, and it shows multiple Bragg reflections having significantly different FWHM, indicating the presence of different crystallographic phases. Phase identification procedure indicates that the sample is mainly composed by Opal-C (95.2%), which is a semi-amorphous hydrated mixture of ordered cristobalite, quartz and tridymite that is usually observed in silica from biogenic origin and lithostatic pressure-induced processes^43–47^. The rest of the sample is composed of $\hbox {Fe}_2\hbox {O}_3$ (4.8%). Since Opal-C is a paracrystalline mixture, it is difficult to determine its lattice parameters, but the values for cristoballite: a = b = 4.97Å and c = 6.92Å can be used as a good approximation. Figure 1C depicts the selected area diffraction (SAD) of DE and diffraction from the 101, 111 and 112 planes of cristobalite can be seen confirming the same structural parameters found through XRD.

Figure 1D and E display results of STEM-EDX analysis on different fragments of milled frustules. The main elements are Si and O, and trace amounts of Fe, Ca and Al were also detected. Figure 1D shows the presence of well defined Fe nanodomains, which confirms the presence of $\hbox {Fe}_2\hbox {O}_3$ in the frustules in addition to $\hbox {SiO}_2$. In contrast, Fig. 1E displays the longitudinal rib of a diatom where the intricate nanostructure of a diatom particle can be seen.

### Electrochemical performance of diatomaceous earth $\hbox {SiO}_2$ anodes

**Figure 2:**
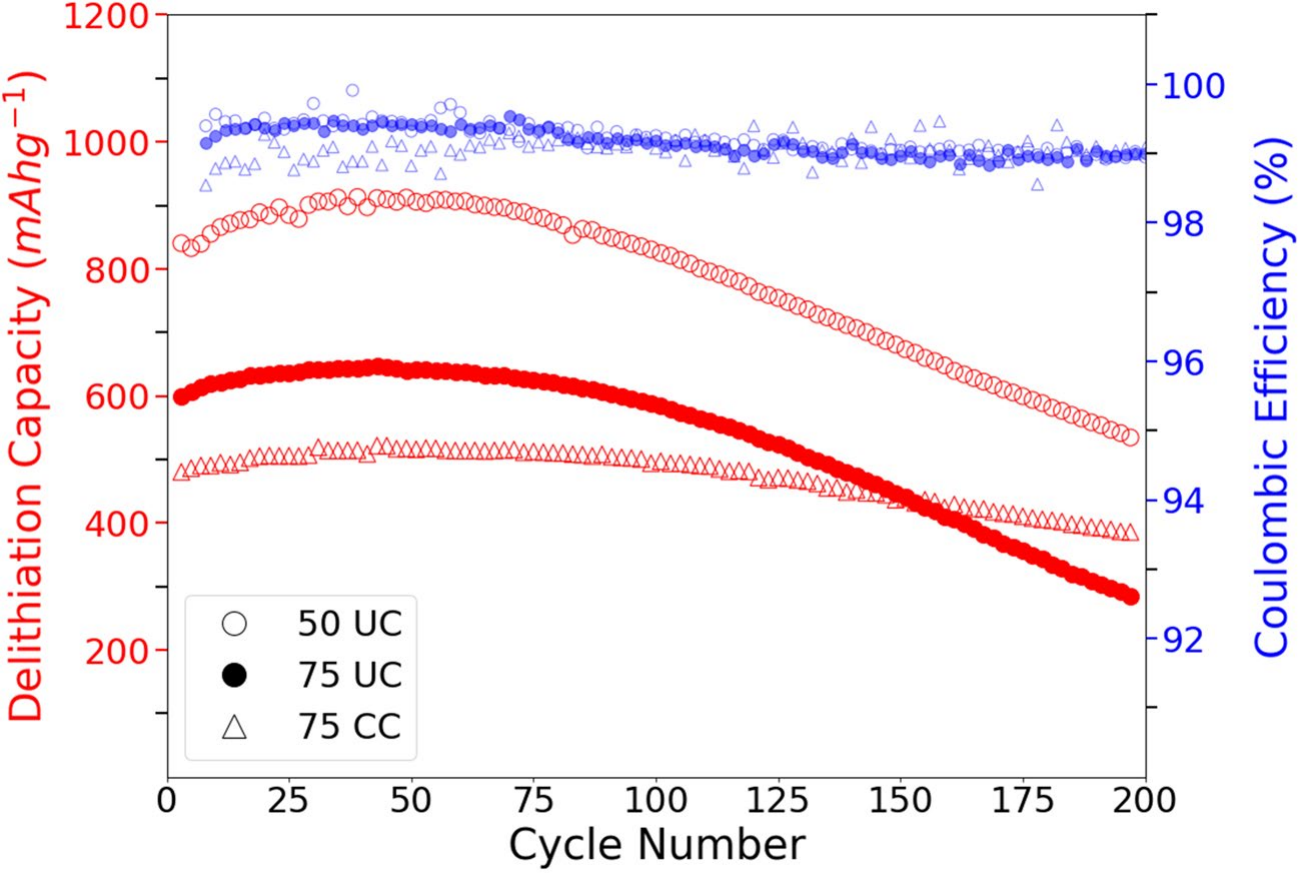
Variation of capacity and CE against cycle number for each electrode composition.

Three different electrode types were fabricated with one composed of 50 wt% diatoms and the other two containing 75wt% of either pure or carbon-coated diatoms. The abbreviations used for each electrode type, (50 UC, 75 UC and 75 CC), and fabrication methods can be found in Table 1 of the "Methods" section. Figure 2 depicts both the evolution of coulombic efficiency (CE) and cycling capacity for all electrode compositions for 200 cycles after five activation cycles performed as described in the Methods section. The specific cycling capacity of the electrode is based on the total mass of the active material which includes the mass of any carbon-coating involved. Thermogravimetric analysis was conducted, Fig. S1 in the supplementary material, to determine the mass of carbon-coating to be 13.7% of the active material. The CE displayed by all electrodes were stably around 99% for all cycles indicating good reversibility of the lithiation reaction. The 50 UC electrode exhibits a considerably higher initial specific capacity of ${840}\,\hbox {mAhg}^-1$ when compared to the other two electrode compositions. 75 CC and 75 UC electrodes contain the same amount of conductive additive but differ on the coating of the particles. They displayed initial specific capacities of ${480}\,\hbox {mAhg}^-1$ and ${600}\,\hbox {mAhg}^-1$, respectively.

Three distinctive regions can be distinguished from the capacity against cycle number plots for all cycled electrodes. Initially, a continuous increase in capacity with cycle number is observed for the first 50 cycles. This initial capacity increase occurred at a similar proportion of about $\approx$ 6.5-8% up until 50 cycles, where maximum values of ${903}\,\hbox {mAhg}^-1$, ${639}\,\hbox {mAhg}^-1$ and ${518}\,\hbox {mAhg}^-1$ are reached for the 50 UC, 75 UC and 75 CC electrodes, respectively. Beyond 50 cycles, the specific capacities remain briefly stable at their maximum values before starting to decay at an increasing rate. At 100 cycles, both 50 UC and 75 UC electrodes display an almost linear capacity fade with a similar rate of $\approx$ ${-150}\,\hbox {mAhg}^-1$ every 50 cycles up to 200 cycles. A much more limited capacity fade is observed for 75 CC electrodes with a relatively low rate of capacity fade at $\approx$ ${-50}\,\hbox {mAhg}^-1$ every 50 cycles up to 200 cycles. Hence, 75 CC electrode displays the highest capacity retention (66%) after 200 cycles, followed by 50 UC (63%) and 75 UC (47%).

**Figure 3:**
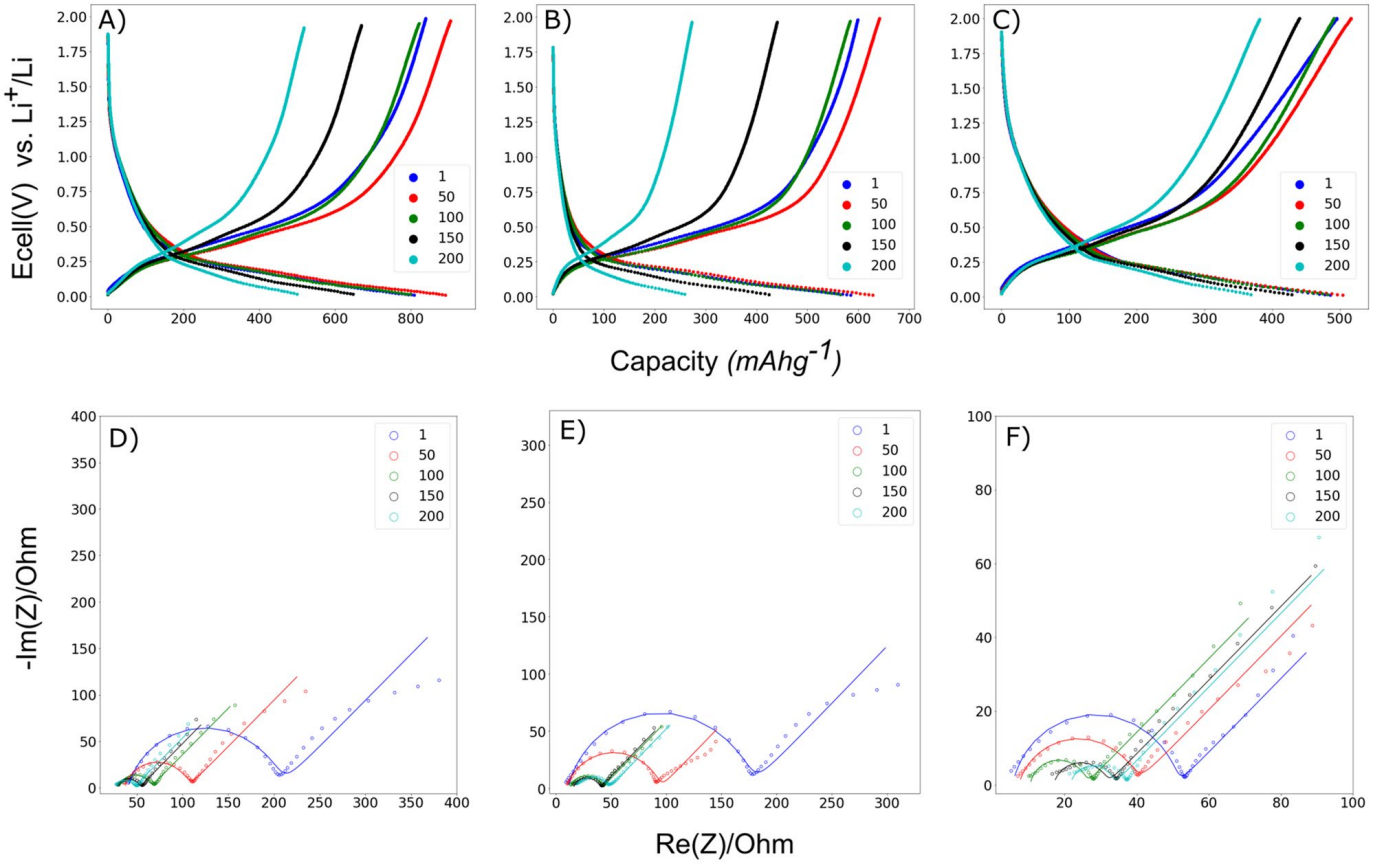
Voltage profiles at cycles 0, 50, 100, 150 and 200 for: (**A**) 50 UC, (**B**) 75 UC and (**C**) 75 CC electrodes. EIS spectras at specific cycle for: (**D**) 50 UC, (**E**) 75 UC and (**F**) 75 CC.

Voltage profiles corresponding to 50 UC, 75 UC and 75 CC electrodes for cycles 1, 50, 100, 150 and 200 are presented in Fig. 3A–C respectively. The voltage profiles of 50 UC and 75 UC electrodes exhibit comparable profiles at all stages of their cycling lifetimes. One important note is that in the first charge/delithiation of the 75 CC electrode, a less inclined profile is observed at higher potentials, which can be attributed to a greater resistance to alloying between Li and Si caused by the carbon coating. At 50 cycles, the delithiation/charge curve has shifted to a lower potential together with a simultaneous increase in discharge capacity. Between cycles 50 to 100, capacity starts to fade and after 100 cycles it is equivalent to their initial values for all cases. Further cycling until 200 cycles results in a marked decrease of the discharge potential with cycle number, which is indicative of increasing electrode polarization. The degree of lithiation at this stage is significantly more shallow and this decrease is most prominent for the 75 UC electrode, and least pronounced for 75 CC electrode, suggesting that the carbon-coating would reduce the build up of internal resistance within the electrode.

Figure 3D–F depicts EIS measurements conducted at 50mV. The cells were held for five hours before conducting EIS measurements at this potential to allow for reaching steady state conditions. The impedance data consists of a single depressed semi-circle in the high frequency region, which has been related to the charge transfer resistance, and a sloped line in the low frequency region. During the initial 50 cycles, a significant reduction in the charge transfer resistance $R_{CT}$ is observed for all electrode configurations. This is in good agreement with the trend in cycling capacity, where a gradual increase in cycling capacity before reaching a maximum after 50 cycles is observed. A further reduction in $R_{CT}$ is observed in the subsequent 50 cycles despite the gradual reduction of the discharge capacity. In the final 100–200 cycles, where the reduction in cycling capacity is substantial, the impedances remain relatively stable with only a slight increase in the bulk resistance evident by a shift of the semi-circle towards the right. A linear fit of the inverse frequency against the imaginary component of the impedance was performed, as shown in Fig. S5 of the supplementary material, and the slope was used as an indication of the double layer capacitance. The double layer capacitance against cycle number is plotted in Fig. S6 which reveals an exponential decay between the double-layer capacitance as cycling proceeds. The double-layer capacitance can be linked to the electrochemical-active surface area in the electrode, suggesting a morphological change in the form of loss of surface area within the electrode.

### Evolution of particle and electrode morphology upon cycling

**Figure 4:**
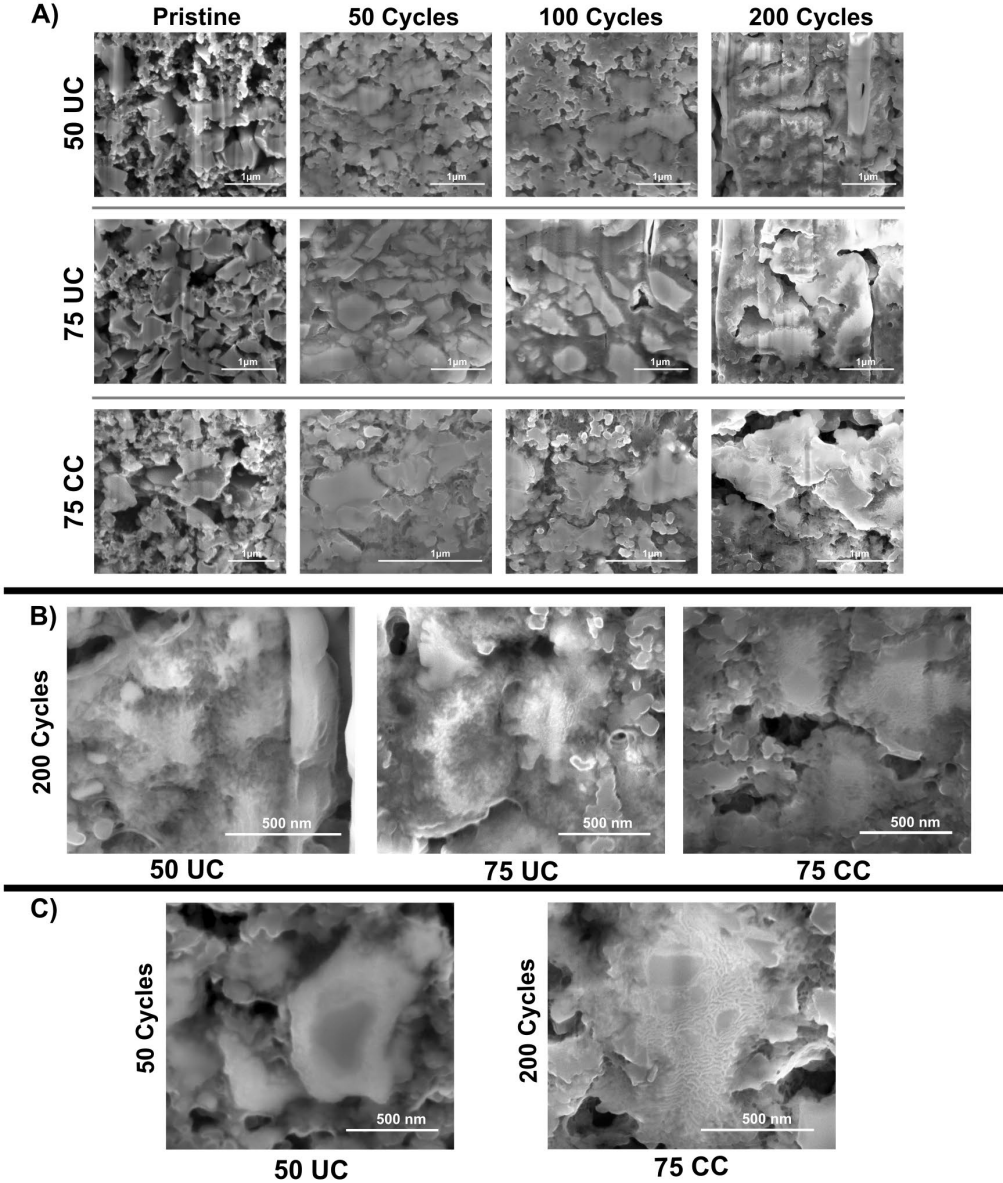
(**A**) FIB-SEM cross sections at electrode level of each electrode composition at pristine condition before activation, 50, 100 and 200 cycles. (**B**) Magnified SEM images of each electrode composition at 200 cycles. (**C**) Images of diatom particles of 50 UC at 50 cycles and 75 CC at 200 cycles highlighting the presence of distinct domains within the electrode.

A study of the electrodes’ morphological evolution upon cycling was conducted using FIB-SEM inspections. Figure 4A depicts morphological changes of the diatom particles within the electrodes at different stages of cycling. The pristine electrodes display a porous matrix of uniformly dispersed solid silica particles with well-defined edges, which appears to be in good contact with the conductive additive. After 50 cycles, where the cycling capacity reaches a maximum, a loss of electrode porosity is observed for all electrode compositions. This loss in porosity is more significant for 75 UC and 75 CC than the 50 UC electrode. At cycle number 100, a further reduction in porosity is observed and there appears to be electrochemical fusion of the active diatom particles. Development of small micro-cracks along the diatom particle edges has also commenced. At 200 cycles, there is a noticeable increase in the roughness of the particles’ surface due to the growth of filigree-like structures. The degree of particle electrochemical fusion is further increased, and the distinction between the active $\hbox {SiO}_2$ diatoms and surrounding conductive matrix is hard to identify. A close-up view of individual active $\hbox {SiO}_2$ diatoms of each electrode composition after 200 cycles can be seen in Fig. 4B. The fracture and cracking of the particle edges at high cycle numbers is more clear in these images. The development of cracks is most significant for electrodes with uncoated DE particles, in which the original particles appear to be completely disintegrated. More magnified images of the electrode particles at different stages of cycling can be found in Fig. S7 in the supplementary materials. In Fig. 4C, what appears to be semi-reacted diatom particles can be seen for 50 UC at 50 cycles where a high contrast between the core and shell of a diatom frustule particle is observed. This could indicate the presence of domains of reacted and pristine $\hbox {SiO}_2$ where the shell consists of $\hbox {SiO}_2$ with a history of lithiation reaction while the core is pristine $\hbox {SiO}_2$. Similar discrepancy in particle morphology can also be observed for 75 CC at 200 cycles, and smooth islands of what appears to be unreacted $\hbox {SiO}_2$ can be seen in a larger electrochemically fused $\hbox {SiO}_2$ particle.

**Figure 5:**
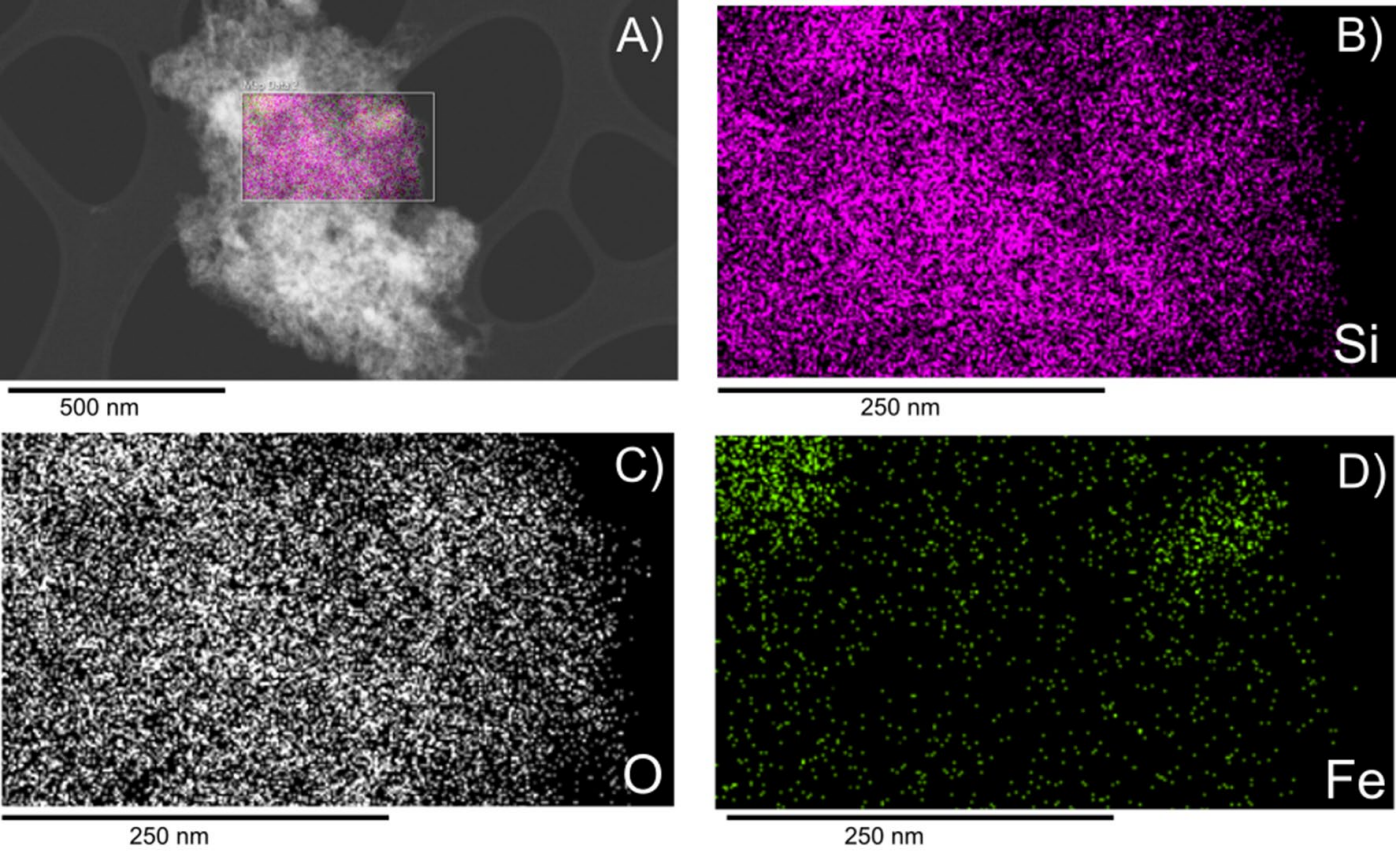
STEM EDX of a 75 UC particle after 200 cycles. (**A**) depicts the overall particle and the layered EDX mappings in the selected region. Individual mappings for (**B**) Si, (**C**) O and (**D**) Fe are also shown.

By isolating the cycled diatom particles from the surrounding conductive and binder matrix, a closer inspection of the individual particles can be achieved. Figure 5 depicts a STEM image and the EDX mappings of a 75 UC electrode particle after 200 cycles. From the comparison between Figs. 1A and 5A, it can be clearly seen that the structural integrity of the particle has been completely lost. From Fig. 5B and C, Si and O appear to be homogeneously distributed throughout the entire particle. From Fig. 5D, the iron in this particle could be found in isolated domains similar to the pristine diatom frustules. The presence of these chemical elements in this configuration suggests that this particle was a diatom particle after repeated electrochemical reaction.

**Figure 6:**
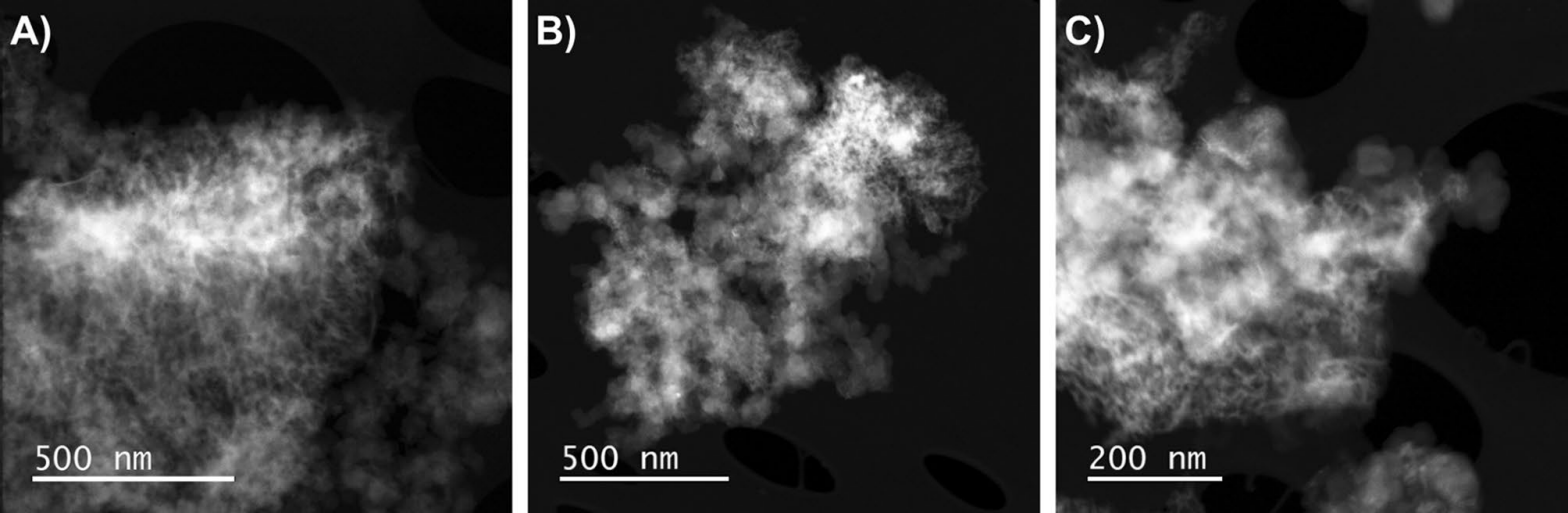
HAADF-STEM of diatom active particles after 200 cycles obtained from (**A**) 50 UC electrode (**B**) 75 UC electrode, (**C**) 75 CC electrode.

Particle morphology of isolated diatom particles after 200 cycles for each electrode composition are shown in Fig. 6. From the images, the degradation state of cycled $\hbox {SiO}_2$ particles can be easily compared. Although all samples show evidence of particle fragmentation and disintegration, with the growth of thread-like structures from the particle edges, non-coated particles show a higher degree of degradation. These results further emphasize FIB-SEM micrographs observations displayed in the previous section.

## Discussion

Though the presence of trace metals in diatoms have been reported before, their presence as concentrated nanodomains in the $\hbox {SiO}_2$ matrix has to the authors’ knowledge not been reported before. The presence of concentrated Fe domains, as shown in Fig. 1D, is slightly unexpected as the other trace metals of either Ca or Al are uniformly distributed throughout the entire diatom particle. However, these occur at extremely low quantities to the point that they remain undetectable by synchrotron XRD. All of the trace metal elements are most likely present in the form of metal-oxides and have been previously found in the siliceous cell walls for both cultured and DE frustules^48^. They are potentially taken up from the environment by living diatoms to perform metabolic functions like photosynthesis or protein production^48–50^.

From Fig. 2, an increase in specific capacity with higher carbon black content was observed. This could be explained as a higher percentage of conductive carbonaceous material would contribute substantially towards electrical conductivity through the electrode, which compensates for the insulating nature of $\hbox {SiO}_2$ frustules. Increased active material reaction throughout the entire electrode can therefore be expected. The slightly lower capacity of 75 CC compared to 75 UC can be due to the fact that despite of the beneficiary effects of increased electronic conductivity provided by the carbon-coating, it can also act as an additional mass-transport barrier that would limit Li-ion diffusion. In addition, including the mass of carbon-coating in the active material reduces the actual mass of electrochemically active $\hbox {SiO}_2$ participating in the reaction.

The initial capacity increase until cycle 50 stems from the conversion of silica to high-capacity silicon, which has been evidenced by differential capacity plots as shown in Fig. S2 of the supplementary material and was also observed in studies by Renmann et al.^51^. Despite the low cut-off voltage, significant presence of the formation of the hard crystalline $\hbox {Li}_15\hbox {Si}_4$ evidenced by a sharp peak at $\approx$0.5V could not be found in the differential capacity plots in Fig. S2. A slight peak at 900mV can be found for all electrodes can be found from cycle 1 to 200, indicating a constant formation or regeneration of the SEI layer. This could be the cause for a lower coulombic efficiency at around 99% and a contributor towards the steady capacity fade through electrolyte depletion by continuous SEI formation. Similar trends on the effect of carbon-coating on capacity fade has been observed by Casino et al.^52^, where the aging mechanism of coated and uncoated silicon thin films anodes were studied. By looking at the differential capacity plots, they attributed the capacity fade to the loss of silicon active material due to electrical insulation as a consequence of volume changes. An increase in capacity retention from 63 to 87% after 100 cycles and 66 to 47% after 200 cycles with the introduction of a carbon-coating was also observed. The additional resistance of the carbon layer and Si/C interface layer further controls the lithiation depth in Si which can improve the cyclic stability by reducing the structural damage of the material^53^. Reduction of particle structural damage could help preserve SEI integrity and maintianing a stable SEI reduces contact between electrolyte and the active material which could improve cycling stability by limiting electrolyte degradation^54,55^.

The EIS data was fitted according to an equivalent Randles circuit to primarily model the charge-transfer contributions. Taking into account additional contributions of the SEI and contact resistance at the electrode/current collector interface would require significantly more complex model. Furthermore, the deconvolution of the spectra to their respective contributions would require additional hypotheses or experiments to ensure the results. Similar trends in evolution of EIS have been observed before in silicon/carbon composite electrodes and the initial reduction in impedance has been attributed to an increase in pressure within the cell as the silicon initially expands^56–58^. The reduction in $R_{CT}$ can be further linked to a continuous conversion of $\hbox {SiO}_2$ to amorphous Si during the first 100 cycles, providing more active material and leading to an increase in both the electric and ionic conductivity within the electrode. The increase in impedance at high cycle numbers has been attributed to electrolyte degradation and blocking of electrolyte channels due to microstructural changes in the electrode and possible electrochemical fusion of Si particles^58^. A linear fit of the $1/\omega$ against the imaginary component of the impedance was done and the slope was used as an indication of the double layer capacitance value. The change in this capacitance against cycle number can be found in Fig. S6 of the supplementary materials and an exponential decay relationship between the capacitance and the number of cycles was found. The double-layer capacitance can be linked to the electrochemical-active surface area in the electrode, suggesting a loss of electrochemically active surface area due to morphological changes within the electrode.

From Fig. 4, the carbon-coating appears to be able to limit the degree of particle degradation to some extent. The fragmentation of active material might lead to particle isolation and poor contact within the electrode, and is a degradation mechanism for reduction in cycling capacity. To the author’s knowledge, morphological studies of electrodes at such high cycle numbers has not been done in $\hbox {SiO}_2$-based anodes before, but similar trends in evolution of the electrode and particle morphology have been observed in silicon and silicon-carbon composite anodes^59,60^. This morphology evolution can be explained by the possible sintering of the Si particles during prolonged cycling together with the intrinsic dealloying process between Li and Si resulting in large agglomerated particles with distinctly cracked and fractured surface^60,61^. A similar degradation mechanism could evidence that a conversion from $\hbox {SiO}_2$ to Si has occurred. The fragmentation of the particle surface could stem from the silicon dealloying process during delithiation and is presumably described as a phase separation process between the Si and Li at the electrode/electrolyte interface as described by Erlebacher et al.^62,63^. Furthermore, the development of microcracks has been associated to anisotropic lithiation, due to the presence of defects, and the asymmetric expansion of silicon, lead to initial crack formation^64,65^. Small initial microcracks provide path for fast lithium-ion diffusion resulting in further non-uniform lithiation and propagation of already formed cracks. The cumulative effect of this mechanism has been associated with the final formation of the thread-like lithiation patterns in Si-matrices. The presence of lithium diffusion paths through the formation of microcracks could also help facilitate the reduction of charge transfer resistance during the first 50 cycles as shown in Fig. 3D–F, especially for the electrodes containing uncoated diatoms. The presence of isolated iron domains as shown in Fig. 5D is very similar to those observed in the pristine diatoms. The preservation of these domains indicates that Fe does not participate in the electrochemical reactions as some loss in structural integrity is expected after electrochemical alloying. This can also be observed in Fig. S8 of the supplementary materials. No signature from iron reaction could be observed from the voltage profiles as well. However, due to the trace amount of Fe present in the diatoms, the actual detection of such reactions remains challenging. From Fig. 6 it is quite evident that the disintegration of the particles is more significant for 50 UC and 75 UC electrodes when compared to 75 CC. This indicates that the carbon-coating is able to effectively mitigate electrode degradation by preserving some of the original particle structure and correlates well with capacity and EIS trends found in the electrochemical results.

## Methods

### Materials characterization

Ex-situ synchrotron X-ray diffraction data of the pristine Diatomaceous earth (DE) $\hbox {SiO}_2$ frustules (Sigma-Aldrich) was collected at Beamline ID31 in the European Synchrotron Radiation Facility (ESRF) using a Pilatus Dectris 2M CdTe detector. The beam energy was at 68keV. Data was reduced and processed using pyFAI package^66^. Identification of crystalline substances was performed by Match! software package of Crystal Impact. Transmission electron microscopy (TEM) analysis was conducted in a JEOL-JEM 2100F multipurpose 200kV field emission analytical electron microscope. Energy-dispersive X-ray spectroscopy elemental maps were obtained using the Oxford X-Max 80 SDD EDX detector (nominal solid angle 0.23 sr). TEM selected area diffraction (SAD) analysis was performed to identify crystalline domains within diatom frustules and HAADF-STEM imaging was performed with a short camera length to promote Z-contrast.

### Electrode preparation & electrochemical characterization

The particle size of the frustules was first reduced through wet ball-milling according to a procedure reported by Renman et al^51^. Carbon-coating of diatom frustules was performed following the method described by Blanco et al.^39^. A slurry solution was then prepared by mixing diatom-based active material, ($\hbox {SiO}_2$ or $\hbox {SiO}_2$/C), carbon black conductive additive (Timcal, C-NERGY C65) and a Na-alginate(Sigma Aldrich) water-soluble binder. The binder solution was prepared separately by dissolving Na-Alginate in deionized water. Slurries for three different electrode compositions were prepared:

**Table 1:** Overview of different compositions of electrode fabricated and their notation for this study.

Notation	Active material (AM)	Carbon-black (CB)	Binder (BI)
50 UC	50 wt% $\hbox {SiO}_2$	35 wt%	15 wt%
75 UC	75 wt% $\hbox {SiO}_2$	15 wt%	10 wt%
75 CC	75 wt% $\hbox {SiO}_2$/C	15 wt%	10 wt%

All slurry solutions were homogenized in a steel jar containing a steel ball in a radially oscillating mixer (RETSCH MM400) at 25Hz for 45 minutes. The slurries were then tape casted on a 18$\upmu \hbox {m}$ thick Cu foil (Schlenk). After allowing for evaporation of excess water at room temperature, the casts were dried in a convection oven at 60$^\circ$C. The casts were then punched using a purpose-built perforator (Hohsen) to obtain circular electrodes with diameter of 16mm. The resulting electrodes have a thickness of $\approx$ 10$\upmu \hbox {m}$ and AM loading of $\approx$ 0.55mg $\hbox {cm}^-2$ for 75 wt% AM electrode and $\approx$ 0,18mg $\hbox {cm}^-2$ for the 50 wt% AM electrode. The electrodes were dried under active vacuum at 120$^\circ$C overnight before being stored in Ar-filled glovebox. They were then assembled into stainless steel coin cells (2032, Hohsen) with lithium foil (Sigma Aldrich) as the combined counter- and reference electrode and a thin porous membrane separator (Celgard 2400). Each cell contained 50$\upmu \hbox {L}$ of electrolyte solution 1M $\hbox {LiPF}_6$ dissolved in (1:1 v/v) solution of ethylene carbonate (EC) and diethyl carbonate (DEC).

Electrochemical studies were carried out in a BCS 805 multi-channel potentiostat (Bio-Logic). All cells are first cycled for five activation cycles at a current density of 50$\hbox {mAg}^-1$, based on the mass of AM, with a potentiostatic lithiation step at low voltages between each cycle. The detailed procedures and reason behind this step are as described in Renman et al. and is referenced to as the **activation cycles** in this work^51^. Galvanostatic cycling with potential limitations was then carried out at 100$\hbox {mAg}^-1$ between 2V and 2mV *vs.* $\hbox {Li}^+$/Li for 200 cycles. EIS was conducted after 50, 100, 150 and 200 cycles of galvanostatic cycling using frequencies of 10mHz to 10kHz with an amplitude of 10mV. EIS measurements were conducted at a potential of 50mV and all cells were held at this potential for five hours to allow for conducting EIS measurements at steady state conditions. The obtained EIS data was fitted using Impendance.py package to equivalent Randles circuit model with a constant phase element replacing the double layer capacity^67^.

### Post-mortem characterization

For post-mortem analysis, cycled coin cells were first decrimped in an Ar-filled glovebox. The electrodes were then extracted and washed with dimethyl carbonate (DMC) to remove any remaining electrolyte salt before being dried under active vacuum until all trace of DMC is removed. Post-mortem morphological studies were performed by FIB-SEM (FEI Helios NanoLab DualBeam FIB). Cross sections were obtained by tilting the sample electrodes to a working angle of 52$^\circ$ before cutting rectangles with side lengths of roughly 20$\upmu \hbox {m}$. The surface of interest was then “polished” by cutting a cleaning cross section pattern along the edge at a lower current. Images were taken with the attached SEM in immersion mode using an acceleration voltage of 5kV and a current of 0.17nA. Post-mortem TEM characterization was also performed. For this, the material was scraped off cycled electrodes and then immersed into an ethanol solution before rigorous ultrasonification for half an hour. The solution was then carefully dripped onto a Cu TEM-grid coated with an amorphous holey carbon film.

### Supplementary Information

Supplementary Information.

## Data Availability

The datasets used and/or analysed during the current study are available from the corresponding author on reasonable request. To the best of the authors’ knowledge, no conflict of interest, financial or other, exists.
