# Supplementary material for: Insights on microstructural evolution and capacity fade on diatom SiO2 anodes for lithium-ion batteries
Source: Sci Rep. 2023 Nov 22;13:20447. doi: 10.1038/s41598-023-47355-7 (PMC10665416; doi:10.1038/s41598-023-47355-7)
Supplement: Supplementary file 1 — Supplementary Information. [file 41598_2023_47355_MOESM1_ESM.docx]

# Supplementary Information

The supporting information contains, thermogravimetric analysis (TGA) data and additional electrochemical results obtained from galvanostatic and Electrochemical Impedance Spectroscopy (EIS) studies and additional FIB-SEM and S(T)EM images.

In **Figure S1**, the TGA data of carbon-coated diatoms is shown, and an onset of mass loss is observed at around 460°C resulting in a loss of 13.7% of the total mass. The mass remained stable despite further increase in temperature.


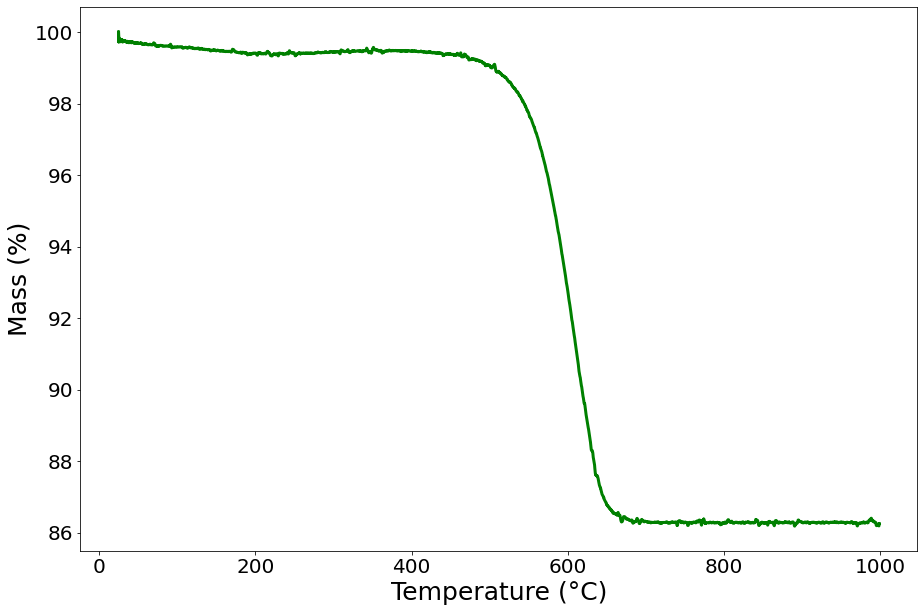


**Figure S1** TGA analysis of carbon-coated diatoms. The green line represents the percentage variation of mass against temperature which is given by the grey line. A loss of 13.7% of the total mass is noticed.

In **Figure S2**, the evolution of the differential capacity at different cycles for each electrode configuration is shown.


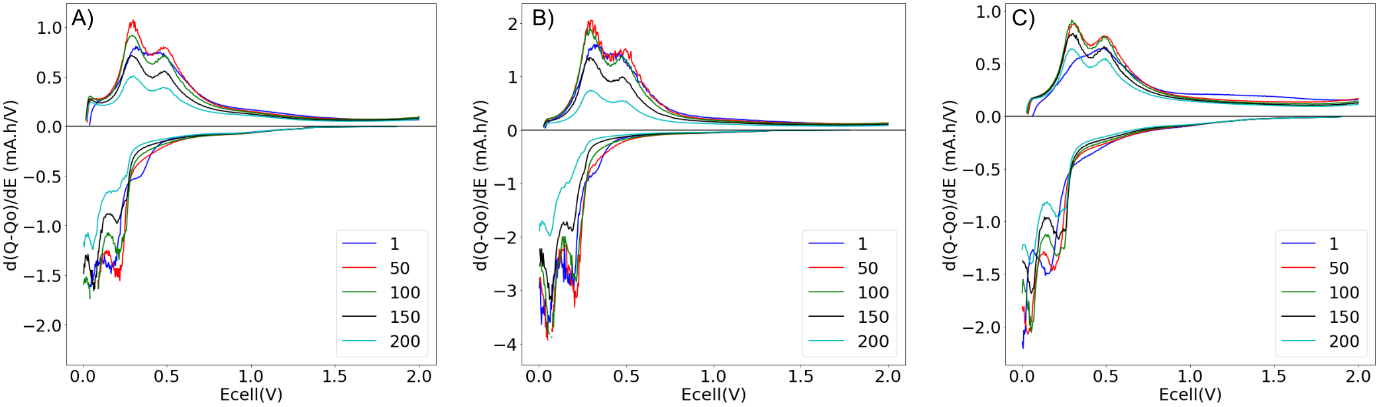


**Figure S2** Differential capacity plots for A) 50 UC B) 75 UC and C) 75 CC electrodes at different cycle numbers. The development of features at voltages associated with the reaction between Li-ions and silicon can be seen as shown in Renman et al ^1^ .

**Figure S3** shows the differential capacity plot of each cell during the first initial formation cycle and the first cycle after formation. Reductive peaks seen in the first formation cycle around 1.5V and 0.9V has been linked to the reduction of EC in the liquid electrolyte and the formation of a stable SEI layer. From Figure S3, these peaks are all significantly reduced between the two cycles. However, in Figure S2, the reductive peak at 0.9V still remains slightly detectable from cycle 1 to 200 for 50UC and 75CC electrodes, and 75 UC to a lesser extent, pointing to new formation or regeneration of SEI.


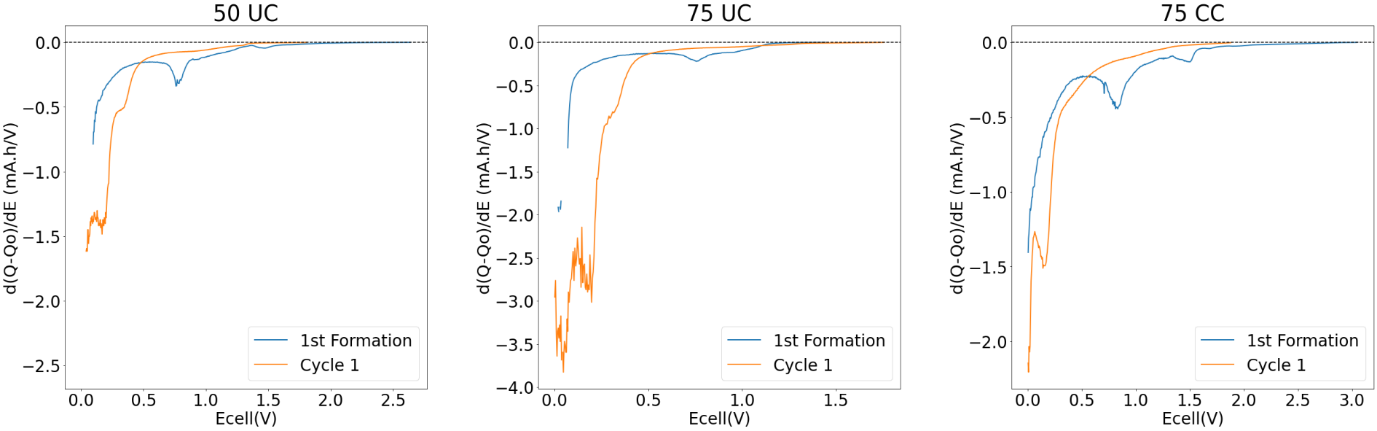


**Figure S3** Differential capacity plots for the first formation cycle for each electrode type.

**Figure S4** depicts the equivalent Randles circuit that the electrochemical impedance spectroscopy data has been fitted against. A simpler Randles Circuit was chosen as only one semi-circle was observed. A constant phase element was chosen as the semicircle was slightly depressed. **Tables 1**, **2** and **3** show the obtained values for each circuit element after fitting for 50 UC, 75 UC and 75 CC electrodes respectively.


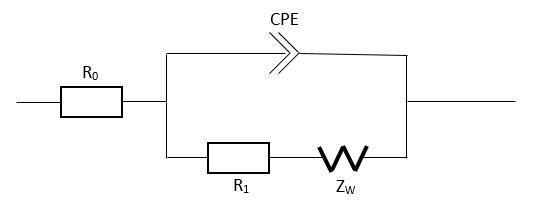


**Figure S4** Figure depicting the equivalent circuit used for fitting of the Randles Circuit. Labels for each of the circuit elements used for the fitting are also given in the figure above.

| 50 UC | | | | | |
| --- | --- | --- | --- | --- | --- |
|  | **Cycle 1** | **Cycle 50** | **Cycle 100** | **Cycle 150** | **Cycle 200** |
| R_0_ | 40.14 | 36.85 | 30.81 | 29.03 | 27.46 |
| R_1_ | 166.2 | 69.0 | 33.7 | 23.4 | 19.0 |
| Z_W_ | 39.1 | 28.9 | 21.2 | 16.3 | 15.3 |
| CPE_q_ | 2.08e-05 | 3.14e-05 | 3.75e-05 | 3.79e-05 | 2.74e-05 |
| CPE_N_ | 0.83 | 0.85 | 0.87 | 0.87 | 0.89 |

**Table 1** Table of values for each equivalent circuit component for 50 UC electrode at different stages of cycling.

| 75 UC | | | | | |
| --- | --- | --- | --- | --- | --- |
|  | **Cycle 1** | **Cycle 50** | **Cycle 100** | **Cycle 150** | **Cycle 200** |
| R_0_ | 10.8 | 9.19 | 13.0 | 15.7 | 17.3 |
| R_1_ | 165 | 85.2 | 28.5 | 23.9 | 30.9 |
| Z_W_ | 29.7 | 12.4 | 13.1 | 12.2 | 13.3 |
| CPE_q_ | 2.37e-05 | 3.94e-05 | 5.80e-05 | 5.66e-05 | 1.23e-04 |
| CPE_N_ | 0.85 | 0.80 | 0.82 | 0.83 | 0.70 |

**Table 2** Table of values for each equivalent circuit component for 75 UC electrode at different stages of cycling.

| 75 CC | | | | | |
| --- | --- | --- | --- | --- | --- |
|  | **Cycle 1** | **Cycle 50** | **Cycle 100** | **Cycle 150** | **Cycle 200** |
| R_0_ | 5.6 | 7.25 | 10.4 | 17.0 | 21.6 |
| R_1_ | 45.5 | 32.5 | 15.3 | 14.7 | 11.8 |
| Z_W_ | 8.66 | 11.8 | 10.9 | 13.7 | 14.1 |
| CPE_q_ | 2.20e-05 | 6.44e-05 | 4.64e-05 | 4.85e-05 | 2.94e-05 |
| CPE_N_ | 0.88 | 0.83 | 0.91 | 0.86 | 0.91 |

**Table 3** Table of values for each equivalent circuit component for 75 CC electrode at different stages of cycling.

**Figure S5** depicts the linear fit of the imaginary component of the impedance at frequencies above 1000 HZ, against the inverse of the frequency at different cycle numbers. The slope of the fitting was used as an indication of the high frequency capacitance.


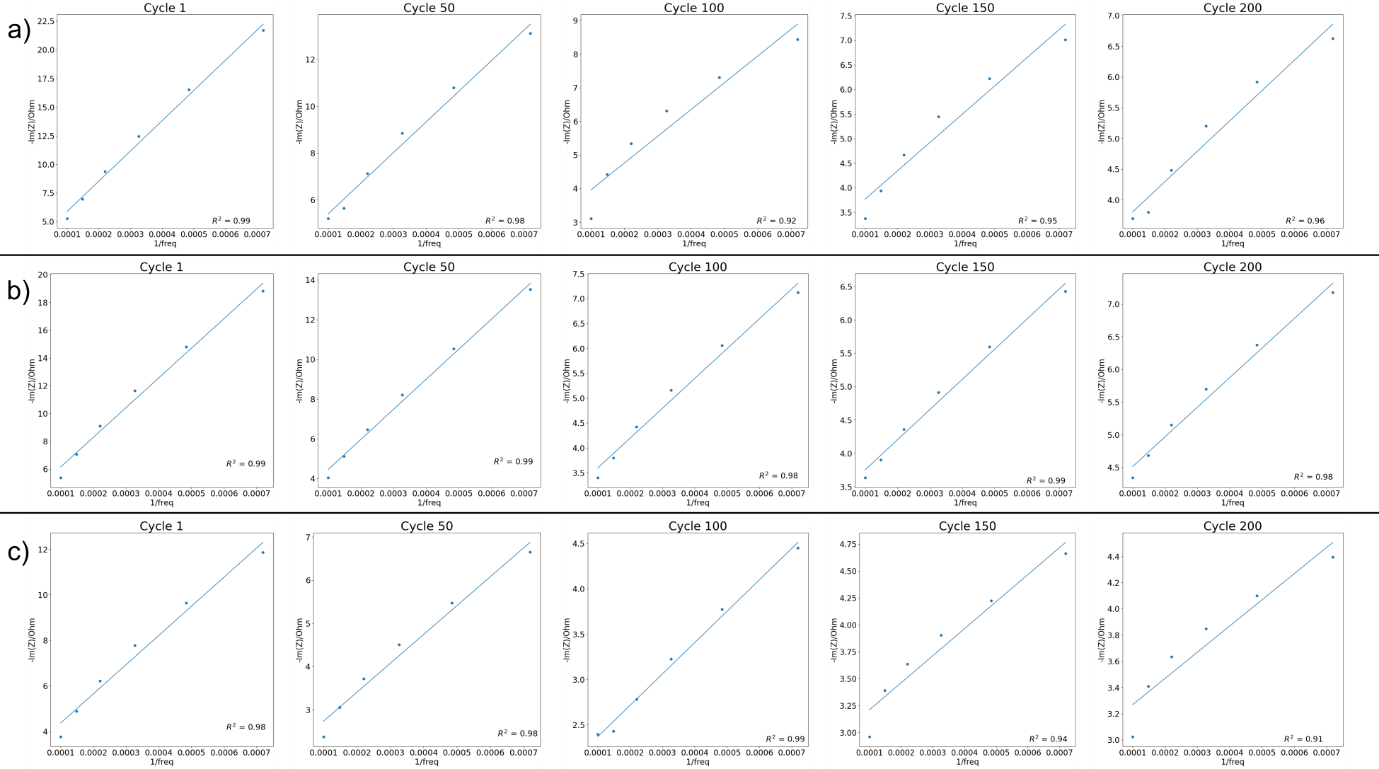


**Figure S5** Linear fitting of the imaginary component of the impedance against the inverse frequency for A) 50 UC, B) 75 UC and C) 75 CC electrodes at different cycle numbers. The capacitance was then obtained from the gradient of the linear fitting.

In **Figure S6**, the evolution of the high frequency capacitance for each electrode configuration was shown. The exponential decay trend of the high frequency capacitance suggests a decrease in electrochemically active surface area as the cycling proceeds.


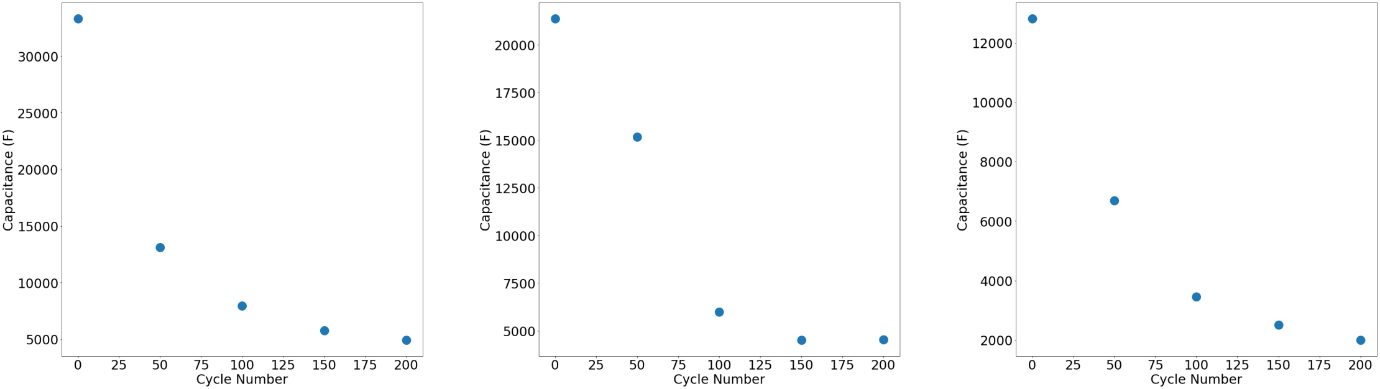


**Figure S6** Variation of Capacitance obtained from EIS with cycle number for A) 50 UC, B) 75 UC and C) 75 CC electrodes at different cycle numbers. The capacitance has been found to decrease exponentially with cycle number.

**Figure S7** depicts more magnified FIB-SEM cross-section images focused on diatom particles at different stages of cycling.


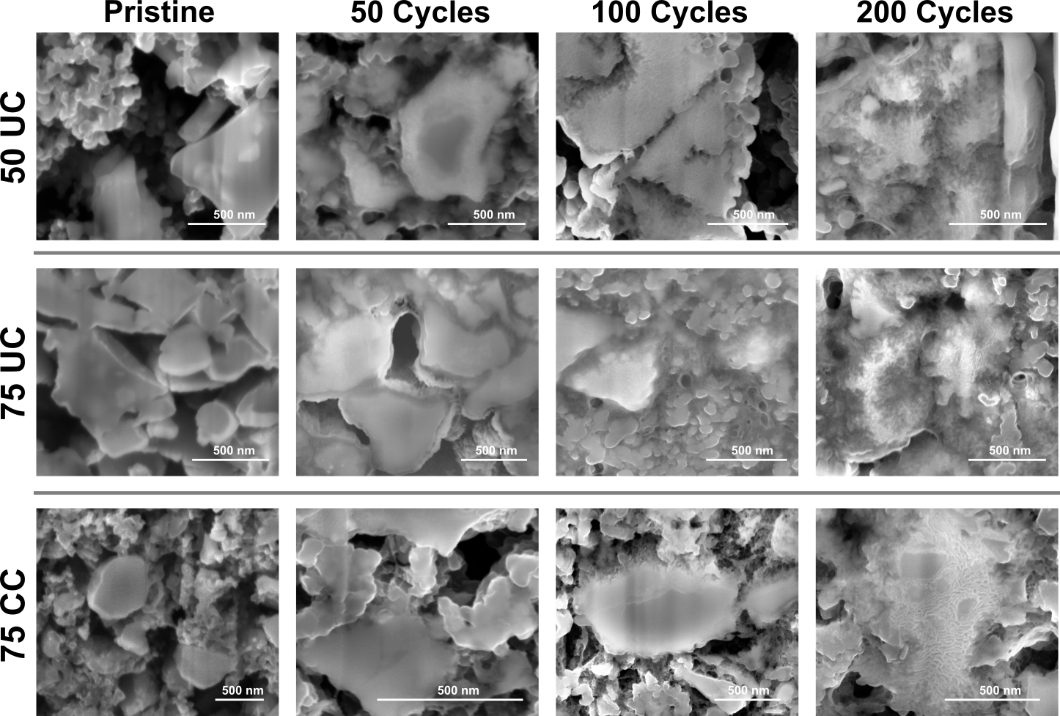


**Figure S7** Magnified FIB-SEM images of the electrode particles at different stages of cycling.

In **Figure S8**, a TEM image of a diatom particle from a 75CC electrode after 200 cycles is shown. As the Fe domains still remains in isolated domains, unlike the Si which has dispersed and expanded after undergoing electrochemical alloying, indicating that Fe does not participate in any electrochemical alloying reaction.


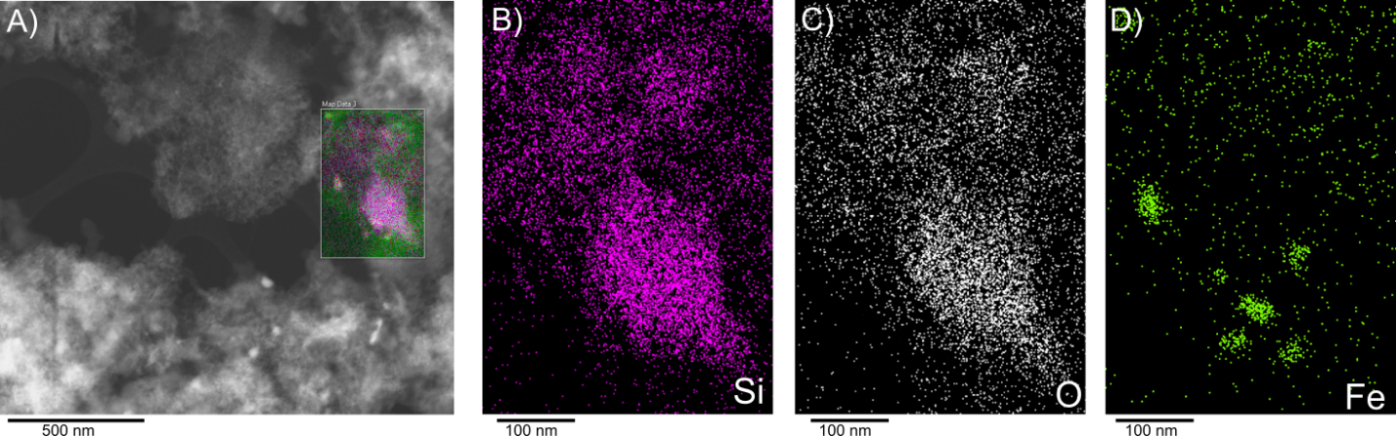


**Figure S8** TEM image of a diatom particle obtained from a 75 CC electrode after 200 Cycles.

# Bibliography

1. Renman, V., Blanco, M. V., Norberg, A. N., Vullum-Bruer, F. & Svensson, A. M. Electrochemical activation of a diatom-derived SiO2/C composite anode and its implementation in a lithium ion battery. *Solid State Ionics* **371**, 115766 (2021).
